# *CRP* and *SAA1* Haplotypes Are Associated with Both C-Reactive Protein and Serum Amyloid A Levels: Role of Suppression Effects

**DOI:** 10.1155/2016/5830361

**Published:** 2016-05-25

**Authors:** Yu-Lin Ko, Lung-An Hsu, Semon Wu, Ming-Sheng Teng, Hsin-Hua Chou

**Affiliations:** ^1^Department of Research, Taipei Tzu Chi Hospital, Buddhist Tzu Chi Medical Foundation, New Taipei City 23142, Taiwan; ^2^The Division of Cardiology, Department of Internal Medicine and Cardiovascular Center, Taipei Tzu Chi Hospital, Buddhist Tzu Chi Medical Foundation, New Taipei City 23142, Taiwan; ^3^School of Medicine, Tzu Chi University, Hualien 97004, Taiwan; ^4^The First Cardiovascular Division, Department of Internal Medicine, Chang Gung Memorial Hospital and Chang Gung University College of Medicine, Taoyuan 33305, Taiwan; ^5^Department of Life Science, Chinese Culture University, Taipei 11114, Taiwan

## Abstract

To test the statistical association of the* CRP* and* SAA1* locus variants with their corresponding circulating levels and metabolic and inflammatory biomarker levels by using mediation analysis, a sample population of 599 Taiwanese subjects was enrolled and five CRP and four SAA1 variants were genotyped. Correlation analysis revealed that C-reactive protein (CRP) and serum amyloid A (SAA) levels were significantly associated with multiple metabolic phenotypes and inflammatory marker levels. Our data further revealed a significant association of CRP and SAA1 variants with both CRP and SAA levels. Mediation analysis revealed that SAA levels suppressed the association between SAA1 genotypes/haplotypes and CRP levels and that CRP levels suppressed the association between CRP haplotypes and SAA levels. In conclusion, genetic variants at the CRP and SAA1 loci independently affect both CRP and SAA levels, and their respective circulating levels act as suppressors. These results provided further evidence of the role of the suppression effect in biological science and may partially explain the missing heritability in genetic association studies.

## 1. Introduction

Acute-phase response is a set of immediate host inflammatory reactions that counteract challenges, such as tissue injury, infection, and trauma. Acute-phase proteins, including C-reactive protein (CRP) and serum amyloid A (SAA), are primarily produced by hepatocytes and chiefly induced by the proinflammatory cytokine interleukin-6 (IL6). In the setting of an acute-phase reaction, CRP and SAA levels can increase up to 100- to 1000-fold for a brief period and typically return to baseline levels within 2 wk [1]. An elevated CRP level has been reported to predict the incidence of cardiovascular events and vascular mortality among apparently healthy individuals and among patients with established cardiovascular disease (CVD) [2–5]. The large JUPITER trial prospectively confirmed that CRP levels aid in tailoring statin treatments for primary prevention in patients with elevated CRP but normal low-density lipoprotein (LDL) cholesterol levels [6]. Family and twin studies have reported additive genetic factors accounting for 27%–40% of the variance in CRP levels [7–9]. Recent genome-wide association studies (GWAS) have identified multiple loci influencing CRP levels, including* CRP* [6, 10, 11].

SAA is secreted by hepatocytes, macrophages, vascular smooth muscle cells, and endothelial cells [12]. SAA promotes the chemotaxis of monocytes and neutrophils and plays critical roles in a wide range of functions including cholesterol transport, high-density lipoprotein (HDL) metabolism, and host defense [13, 14]. Furthermore, SAA induces, promotes, or influences susceptibility to several chronic diseases such as atherosclerosis and its clinical complications [15–18]. Twin studies have suggested a substantial genetic contribution of baseline SAA levels, with heritability estimates of 49%–67% [9]. GWAS have reported that the chromosome region at 11p15.5-p13, which includes the* SAA* family, accounts for most of the explained variance in circulating SAA levels [19]. The* SAA1* single nucleotide polymorphism (SNP) rs12218 was found to be associated with multiple atherosclerotic CVDs and related risk factors [20–23]. Mediation analysis suggested a suppression effect of soluble E-selectin (sE-selectin) on the association between* ABO* genotypes and triglyceride to HDL cholesterol ratios [24]. Gene-centric analysis identified variants associated with pathways shared by different inflammatory biomarkers [25]. Thus, using pathway and mediation analysis is crucial to further elucidating the genetic determinants of inflammatory marker levels, which may be one of the reasons for the missing heritability in GWAS. Therefore, we tested the clinical and biomarker correlates of CRP and SAA levels and the statistical association of* CRP* and* SAA* locus variants with the circulating levels of metabolic and inflammatory biomarkers by using mediation analysis in a Taiwanese sample population.

## 2. Participants and Methods

### 2.1. Study Population

This study was approved by the institutional review board of Taipei Tzu Chi Hospital, Buddhist Tzu Chi Medical Foundation (IRB number: 02-XD38-089). A total of 599 Han Chinese subjects (315 men and 284 women with mean ages of 46.1 ± 10.0 and 46.8 ± 9.9 y, resp.) were recruited during routine health examinations between October 2003 and September 2005 at Chang Gung Memorial Hospital. All of the participants provided written informed consent. The subjects responded to a questionnaire on their medical history and lifestyle characteristics and underwent a physical examination that involved measurement of height, weight, waist and hip circumference, and blood pressure (BP) in the sitting position after 15 min of rest. Fasting blood samples were obtained from each subject. Exclusion criteria included an age younger than 18 y, CRP levels of above 10 mg/L, a history of myocardial infarction, stroke, or transient ischemic attack, cancer, and current renal or liver disease. The clinical characteristics and biometrics of the study population are summarized in Table 1. Hypertension, obesity, and the current smoking status were defined as previously reported [27].

### 2.2. Genomic DNA Extraction and Genotyping

Genomic DNA was extracted as reported previously [24]. Nine SNPs around the* CRP* and* SAA1* loci were selected for this study (see Supplementary Table 1 in Supplementary Material available online at http://dx.doi.org/10.1155/2016/5830361). Genotyping was performed using PCR, followed by restriction enzyme digestion or using TaqMan SNP genotyping assays obtained from Applied Biosystems (ABI, Foster City, CA, USA).

### 2.3. Laboratory Examinations and Assays

The laboratory examinations and assays were performed as described previously [27, 28]. Most markers, including serum CRP, SAA, soluble intercellular adhesive molecule (sICAM1), soluble vascular cell adhesive molecule (sVCAM1), sE-selectin, adiponectin, matrix metalloproteinase-9 (MMP-9), and plasma monocyte chemotactic protein-1 (MCP-1), were measured using a sandwich enzyme-linked immunosorbent assay (ELISA) developed in-house. All in-house kits showed good correlation with commercially available ELISA kits. Circulating serum resistin, lipocalin-2, MMP-2 and plasma MMP-1, soluble P-selectin (sP-selectin), soluble tumor necrosis factor receptor II (sTNFRII), and interleukin-6 (IL6) were measured using commercially available ELISA kits from R&D (Minneapolis, MN, USA).

### 2.4. Statistical Analysis

The chi-square test was used for testing to compare categorical variables of diabetes mellitus and smoking. The clinical characteristics that were continuous variables are expressed as means ± SDs and were tested using a two-sided *t*-test or analysis of variance (ANOVA). Pearson correlation coefficients (*r*) were calculated to determine the association between CRP or SAA levels and clinical and biochemical factors with the adjustment of age and sex. Furthermore, a general linear model was applied to capture the major effect of each polymorphism on clinical and biochemical variables with BMI, age, gender, and smoking status as confounding covariates. We also used dominant models for numeric association test after recoding our SNPs from categorical variables to continuous variables, such as 0, 1 of a particular allele. A value of *P* < 0.05 using two-sided tests was considered statistically significant. All the above calculations were performed with standard statistical SPSS 12 software (SPSS, Chicago, IL, USA). Golden Helix SVS Win32 7.3.1 software was used to analyze the deviation from the Hardy-Weinberg equilibrium and to estimate the linkage disequilibrium between polymorphisms. Values of HDL cholesterol, LDL cholesterol, total cholesterol, triglyceride, CRP, SAA, sICAM1, sVCAM1, sE-selectin, sP-selectin, MMP-1, MMP-2, MMP-9, MCP-1, sTNFRII, and IL6 were logarithmically transformed prior to statistical analysis to adhere to a normality assumption. The Bonferroni method was used to correct for multiple comparisons where applicable. To explore the mediating effects of SAA levels on the relationship between the* SAA1* genotypes/haplotypes and CRP levels, and vice versa for the CRP genotypes/haplotypes, a conceptual model was hypothesized for the test, and four criteria were suggested for evaluating the mediating and suppression effects [24]. For example, in criterion one, independent variable (*SAA1* genotypes/haplotypes) must predict the mediator (SAA levels). In* criterion* two, the mediator must predict the dependent variable when adjusting for independent variable (CRP level). The mediation effect was calculated as the product of the two regression coefficients from* criterion* one and* criterion* two, which reflected the intermediate pathways from independent variable to mediator and in turn to dependent variable. The regression coefficient relating independent variable to dependent variable adjusting for the mediator was expressed as direct effect. For* criterion* three, the total effect, expressed as the effect of independent variable on dependent variable, can be obtained by summation of direct and mediation (indirect) effects. In* criterion* four, the mediation effect must be significant using the procedure outlined by Sobel [26, 29]. A suppression effect may be indicated in a situation when the direct effect is larger than the total effect [30]. In this situation, the direct and indirect effects often have fairly similar magnitudes and opposite signs, which may entirely or partially cancel each other out and result in zero or a nonzero but insignificant total effect [31]. The *β* coefficients and standard errors from the model above were further used to conduct a Sobel test for mediation [32]. The Sobel test was performed using a tool for mediation tests (http://www.quantpsy.org/sobel/sobel.htm), in which the null hypothesis *H*_0_: *αβ* = 0 is tested. The test statistic *S*, which is approximately distributed as *Z* (2), is obtained by dividing the estimated mediation effect (*αβ*) by the standard error (*δ*) in (1). The reported *P* values are drawn from the unit normal distribution under the assumption of a *Z* value of the hypothesis that the mediated effect equals zero in the population. ±1.96 are the critical values of the test ratio which contain the central 95% of the unit normal distribution:

(1) δαβ2=δα2β2+δβ2α2

(2) Z=αβSQRTδα2β2+δβ2α2,

where SQRT is square root.

## 3. Results

### 3.1. Clinical and Biochemical Characteristics

A summary of demographic features, clinical profiles, and biomarker levels for the health examination participants is provided in Table 1. No significant deviation from the Hardy-Weinberg equilibrium was detected for the studied polymorphisms. All of the studied polymorphisms in the same chromosomal region were in strong pairwise linkage disequilibrium (Supplementary Tables 2 and 3).

### 3.2. Association of CRP and SAA Levels with Clinical Parameters and Other Biomarker Levels

Circulating CRP levels were positively associated with approximately all anthropometric and metabolic traits, except for the QUICKI index and HDL cholesterol level, in which a negative association was observed, and the LDL cholesterol and urinary ACR levels, in which no significant association was observed (Table 2). By contrast, SAA levels had a similar but less significant trend of the associations, having no significant association with waist-hip ratios, eGFR, fasting plasma glucose, HDL cholesterol levels, and urinary ACR. Regarding inflammatory markers and adipokine levels, CRP levels were positively associated with circulating SAA, fibrinogen, sE-selectin, sTNFRII, IL6, leptin, and lipocalin-2 levels and negatively associated with adiponectin levels, whereas SAA levels were positively associated with circulating CRP, fibrinogen, IL6, and leptin levels.

### 3.3. Association of CRP and SAA1 Genotypes/Haplotypes with CRP and SAA Levels

After adjusting for clinical covariates, significant association of three* SAA1* polymorphisms with SAA levels was observed using an additive inheritance model (Table 3). Using a dominant inheritance model, a minor allele of rs4638289 was found to be associated with a higher SAA level (*P* = 7.48 × 10^−25^), whereas minor alleles of rs11024591 and rs7131332 were found to be associated with a lower SAA level (*P* = 1.28 × 10^−16^ and *P* = 1.93 × 10^−16^, resp.). Using haplotype analysis, two haplotypes (*GATT* and* AAAC*) inferred from four SNPs were found to be associated with SAA levels (*P* = 4.15 × 10^−28^ and *P* = 1.20 × 10^−27^, resp.) (Table 4). The association between* CRP* genotypes/haplotypes and CRP levels has been reported previously [33]. In this study, we excluded subjects aged younger than 18 y or had CRP levels above 10 mg/L, and the results were similar to those of previous reports, with the exception of no significant association of the rs1800947 genotypes with CRP levels (Table 3).

### 3.4. Association of CRP and SAA1 Genotypes/Haplotypes with Clinical Parameters and Other Biomarker Levels

**Figure 1:**
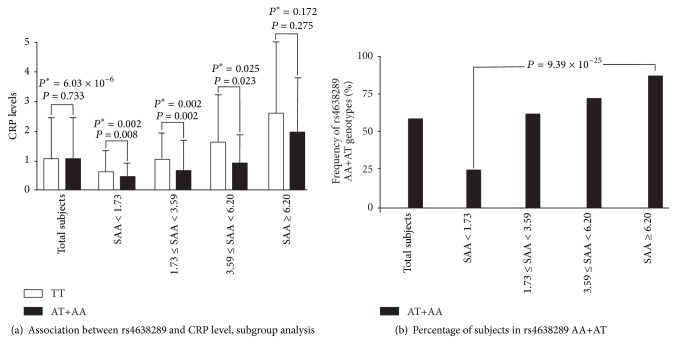
(a) Subgroup analysis with SAA quartiles on the association between rs4638289 genotypes and CRP levels. (b) Frequencies of subjects with* AA*+*AT* genotypes of rs4638289 in each SAA quartile. *P* value: adjusted for age, sex, body mass index, smoking, and medications for hypertension, diabetes mellitus, and dyslipidemia. *P* ^*∗*^ value: further adjusted for SAA levels.

Association of* CRP* and* SAA1* genotypes/haplotypes with various clinical parameters and biomarker levels is shown in Supplementary Tables 4–7. After further adjusting for SAA levels, a significant association of CRP levels with rs4638289, rs7131332, and rs11024591 was observed in the additive inheritance model (*P* = 2.48 × 10^−5^, *P* = 0.040, and *P* = 0.016, resp.) and with rs4638289 in dominant inheritance model (*P* = 2.41 × 10^−5^). Adjustment of circulating SAA and CRP levels revealed that the* SAA1* haplotype* AAAC* was associated with lower CRP levels (*P* = 1.91 × 10^−4^) and that the* CRP* haplotype* AAGCG* was associated with lower SAA levels (*P* = 0.046). Subgroup analysis of SAA quartiles revealed at least a trend of lower CRP levels with the minor allele of the rs4638289 genotypes in each SAA quartile (*P* = 0.008, *P* = 0.002, *P* = 0.023, and *P* = 0.275, separately). By contrast, a significant increase in the minor allele frequencies of the rs4638289 genotypes was observed in the higher SAA quartiles (*P* = 9.39 × 10^−25^), which illustrated no significant association between CRP levels and rs4638289 genotypes when all of the subjects were pooled (*P* = 0.733) (Figure 1).

### 3.5. Mediation Analysis of Suppression Effects

**Figure 2:**
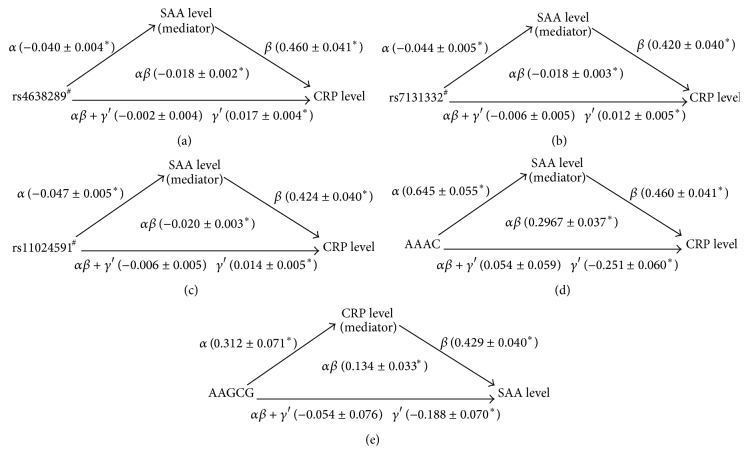
A three-variable mediation model: SAA and CRP levels as a mediator of the association between the* SAA* and* CRP* genotypes/haplotypes and CRP and SAA levels. Linear regression models were used to assess the following path associations: in Figure 1(a): (*α*) relationship between the* SAA* genotype and SAA level, (*β*) relationship between SAA level and CRP level, (*αβ* + *γ*′) relationship between the* SAA* genotype and CRP level, and (*γ*′) relationship between the* SAA* genotype and CRP level after adjustment for SAA levels. Each estimate along the path represents the unstandardized *β* coefficient from the regression model. The results indicate that, after adjustment for SAA levels, the* SAA* genotypes exhibited a stronger association with CRP level. The direct effects (*γ*′) of the* SAA* genotypes on the CRP level (0.017) were greater than the total effects (*αβ* + *γ*′) (−0.002) and revealed similar magnitudes and opposite signs than those of the mediation effects (*αβ*) (−0.018), suggesting significant mediation (suppression) by the SAA levels. All of the models were adjusted for age, sex, BMI, current smoking status, and medications for hypertension, diabetes mellitus, and dyslipidemia. ^*∗*^ *P* < 0.05. In addition, the other analysis exhibited similar suppression effects ((b), (c), (d), and (e)). ^#^ *SAA* genotypes.

Four criteria were applied to establish mediation and suppression effects.* SAA1* and* CRP* genotypes/haplotypes were analyzed (Table 5). In brief, the* SAA1* genotypes/haplotypes were significantly associated with SAA levels (criterion 1), which in turn had significant positive effects on CRP levels (criterion 2). The total effect of* SAA1* genotypes/haplotypes on CRP levels was −0.002, −0.006, −0.006, and 0.054 with all *P* values nonsignificant (criterion 3). Sobel tests for mediation of the results of the corresponding CRP levels showed *z* = 9, 6, 6.67, and 8.02 (all *P* < 10^−8^) (criterion 4). Moreover, the direct effects (*γ*′) of the* SAA1* genotypes/haplotypes on CRP levels were greater than their total effects (*αβ* + *γ*′) and had similar magnitudes as mediation effects but opposite signs (*αβ*), demonstrating a suppression effect in this model (Figure 2). The suppression effects of CRP levels on the association between the* CRP* haplotype* AAGCG* and SAA levels, of the criteria, were also observed.

## 4. Discussion

This investigation involved analyzing the association of* CRP/SAA1* SNPs and CRP/SAA levels with various clinical parameters and biomarker levels. As predicted, both CRP and SAA levels correlated with multiple metabolic phenotypes and inflammatory marker levels, suggesting their crucial roles in atherosclerotic processes. Similar to the results of previous studies, our data showed a strongly significant association between* CRP/SAA1* SNPs and their respective circulating levels. By adjusting their individual circulating levels further, we also found* SAA* and* CRP* variants to be significantly associated with circulating CRP and SAA levels, respectively. Mediation analysis revealed that SAA levels have a suppression effect on the association between* SAA1* genotypes/haplotypes and CRP levels, and CRP levels have a suppression effect on the association between* CRP* haplotypes and SAA levels. These results suggested the relevance of* SAA1/CRP* variants as the genetic determinants of both CRP and SAA levels.

*SAA1 SNP and SAA Levels*. Marzi et al. reported two gene loci, 11p15.5 and 1p31, to have a considerable impact on SAA levels, which compose approximately 20% of the total estimated heritability [19]. In this study, we confirmed a highly significant association of rs4638289 with SAA levels in a Taiwanese sample population. Previous studies have shown that the* SAA1* SNP rs12218 was associated with multiple atherosclerotic CVDs and related risk factors [20–23]. However, the association of rs12218 with SAA levels has not been elucidated previously. Our data showed no evidence of the association of the rs12218 genotypes with SAA levels and various other metabolic or inflammatory phenotypes (Table 3 and Supplementary Table 5). Additional studies may be necessary to elucidate further the role of this SNP in different ethnic populations.

*Association of CRP Variants and CRP Levels with Atherogenesis: Importance of Pathway Analysis*. Compelling experimental and clinical evidence suggests a crucial role of inflammation in the initiation and progression of atherosclerosis [34]. An abundance of epidemiological data has linked circulating inflammatory biomarkers with the risk of atherosclerotic CVDs and their adverse outcomes [35]. Among the wide array of inflammatory biomarkers that have been studied, high-sensitivity CRP has received the most attention for its use in screening and risk reclassification and as a predictor of clinical response to satin therapy [36]. Our study revealed a significant association of CRP and SAA levels with approximately all of the studied atherosclerosis-related traits, including glucose metabolism, renal function, levels of various adipokines, and inflammatory markers, as well as adiposity and BP status. Although most of the correlations were not strong, one possibility is that the study participants are relatively healthy without manifested systemic diseases. Further study in patient populations, such as those with atherosclerotic cardiovascular diseases, will help us to understand whether CRP/SAA levels have a stronger association with various atherosclerotic risk factors in disease populations. These results further support the multifaceted perspective of the CRP and SAA levels affecting the pathogenesis of atherosclerosis. Recent GWAS studies have shown multiple genetic determinants of CRP and SAA levels; however, the probability of SNPs contributing much to the current approach of risk assessment, based on conventional risk factors, is low. Our data provide the first evidence suggesting the importance of* CRP* and* SAA1* variants as genetic determinants of both CRP and SAA levels. Carty et al. showed that gene-gene interaction between rs4638289 and genetic variants of* SAA* regulators influences CVD [37]. Shah et al. revealed the association of interleukin-6 receptor (*IL6R*) genotypes with both IL6 and CRP levels [25]. Thus, a paradigm shift would involve using a gene-centric approach to investigating an entire pathway rather than assessing isolated mutations for providing more useful information on complex traits that involve a high number of genes and are subject to environmental regulation of gene expression and cellular phenotypes [25].

*Suppression Effect and Possible Mechanisms*. Suppression effects have been rarely reported in biological science. Mediation hypotheses suggest a suppression effect if the statistical removal of a mediating effect enhances the relationship between the independent and dependent variables. We previously reported that sE-selectin levels have a suppression effect on the association between* ABO* blood group genotypes and triglyceride to HDL cholesterol ratios [24]; this emphasizes the importance of the relationship between ABO blood groups and atherogenesis. In our recent study, adiponectin levels also acted as suppressors for the association between* CDH13* SNPs and metabolic syndrome and metabolic phenotypes [38]. The data in our current study suggested that SAA and CRP levels act as suppressors of the association between their respective gene variants and circulating levels of other inflammatory biomarkers. The association between* SAA* rs4638289 genotypes and CRP levels became more significant in subgroup analysis with different quartiles of SAA levels. Lower CRP levels were observed in each SAA quartile in subjects with the minor allele A of the rs4638289 genotypes, whereas higher* AA*+*AT* genotype frequencies were observed with increased SAA quartiles (which are associated with higher CRP levels); this may partially explain why no significant difference was observed between rs4638289 genotypes and CRP levels when all of the studied subjects were pooled for analysis without adjusting the SAA levels. Altogether, these findings indicate the crucial role of suppression effects in biological science. Mediation analysis would elucidate the additional genetic determinants of biomarker levels and disease status.

Several possible biological mechanisms of the suppression effect may be considered. MicroRNAs have emerged as key gene regulators, including trans- or cisregulation, for diverse biological pathways in various vascular and metabolic diseases [39]. Online resources for microRNA target prediction may be helpful in searching for possible candidates linked between* CRP* and* SAA* loci. It is also possible that the association of the* SAA* polymorphism with CRP levels may be due to linkage disequilibrium with another polymorphism that is linked to CRP regulation. More importantly, the SAA and CRP levels are affected by multiple proinflammatory cytokines [40]. The proinflammatory cytokine IL6 is a critical mediator that induced both CRP and SAA expression in hepatocytes.* IL6R* gene variants have been associated with CRP levels [25]. Significant associations of circulating IL6 levels with CRP and SAA levels were noted in this investigation; however, our preliminary data revealed that the association of* CRP/SAA* genotypes/haplotypes with CRP and SAA levels was not affected with adjustment of IL6 levels (data not shown). Thus, further investigation involving multiple candidate targets will be necessary in the future to establish the molecular basis of the suppression effect.

*Suppression Effects May Partially Explain the Missing Heritability*. Recent GWAS have identified hundreds of genetic variants associated with complex human diseases and traits. However, complex inheritance can assume numerous forms and GWAS have only partially explained heritability. The missing heritability may be due to multiple factors, including a high number of variants with smaller effects yet to be found, rare variants with larger effects, which are poorly detected by available genotyping arrays, structural variants poorly captured by existing arrays, low power for detecting gene-gene and gene-environment interactions, and sequence-independent epigenetic effects including noncoding microRNAs [41, 42]. Our recent investigations revealed that mediation analysis of suppression effects may elucidate several previously unidentified associations between genetic variants in one gene and other closely associated metabolic or inflammatory phenotypes, which may in turn partially explain the missing heritability in complex diseases.

*Limitation*. Our study has several limitations, one of which is the relatively modest number of studied subjects. Furthermore, only 9 of the* CRP* and* SAA1* SNPs were analyzed, and this incomplete genotyping may not represent all of the haplotypes in* CRP* and* SAA1*. Another limitation of this study is its cross-sectional design, which could draw only limited inference regarding the relationships between exposure and outcome. Finally, the examined subjects were ethnically Chinese; hence, caution should be exercised when extrapolating our results to other ethnic groups.

## 5. Conclusion

Our data revealed a significant association of* CRP* and* SAA1* variants with both CRP and SAA levels, which are highly correlated with multiple atherosclerosis-related traits. These results provide further evidence of the role of suppression effects in biological science and may partially explain the missing heritability in genetic association studies. Further analysis of the interrelationship between entire pathways of inflammatory gene variants and circulating levels may further elucidate the pathogenesis of atherosclerotic CVDs.

## Supplementary Material

Supplementary Materials include tables for the baseline data of the C-reactive protein (*CRP*) and serum amyloid A (*SAA1*) variants, linkage disequilibrium between the *CRP* and *SAA1* variants and the association of the *CRP* and *SAA1* variants with clinical parameters and other biomarker levels.

## Figures and Tables

**Table 1:** Baseline characteristics of the studied subjects.

	Total	Men	Women	*P* value
Number	599	315	284	
Age (years)	46.1 ± 10.0	45.5 ± 10.0	46.8 ± 9.9	0.125
Systolic BP (mmHg)	113.1 ± 16.2	114.1 ± 14.4	112.0 ± 17.9	0.127
Diastolic BP (mmHg)	75.0 ± 10.0	76.9 ± 9.7	73.1 ± 9.9	<0.001
Total cholesterol (mg/dL)	199.0 ± 36.4	201.3 ± 36.9	196.5 ± 35.8	0.108
HDL cholesterol (mg/dL)	55.3 ± 14.3	49.8 ± 12.0	61.3 ± 14.2	<0.001
LDL cholesterol (mg/dL)	116.2 ± 32.9	118.8 ± 33.9	113.3 ± 31.5	0.041
Triglyceride (mg/dL)	142.9 ± 119.5	173.4 ± 148.6	109.5 ± 60.6	<0.001
Body mass index (kg/m^2^)	24.3 ± 3.4	25.0 ± 3.1	23.6 ± 3.6	<0.001
Diabetes mellitus (%)	5.0	5.7	4.2	0.404
Current smokers (%)	19.5	33.7	3.9	<0.001
Fasting plasma glucose (mg/dL)	96.4 ± 22.6	99.1 ± 26.0	93.5 ± 17.7	0.003
Fasting serum insulin (*μ*U/mL)	9.2 ± 4.8	9.8 ± 5.6	8.5 ± 3.7	0.001
HOMA-IR index	2.2 ± 1.4	2.4 ± 1.6	2.0 ± 1.1	<0.001
QUICKI	0.35 ± 0.02	0.34 ± 0.03	0.35 ± 0.02	<0.001
Adiponectin (mg/L)	7.2 ± 5.2	5.5 ± 4.0	9.1 ± 5.7	<0.001
Resistin (ng/mL)	18.5 ± 14.4	17.7 ± 12.0	19.4 ± 16.5	0.137
Lipocalin-2 (ng/mL)	79.9 ± 52.1	83.1 ± 59.8	76.5 ± 42.0	0.104
CRP (mg/L)	1.1 ± 1.4	1.1 ± 1.4	1.2 ± 1.4	0.186
Fibrinogen (mg/dL)	262.6 ± 68.3	260.3 ± 70.3	265.1 ± 66.1	0.389
sE-selectin (ng/mL)	53.2 ± 25.2	59.8 ± 26.0	45.8 ± 22.0	<0.001
sP-selectin (ng/mL)	138.4 ± 115.4	152.9 ± 131.1	122.4 ± 92.8	0.001
SAA (mg/L)	5.3 ± 11.1	5.9 ± 13.3	4.7 ± 8.0	0.403
sICAM1 (ng/mL)	240.0 ± 110.9	241.7 ± 108.9	238.1 ± 113.2	0.730
sVCAM1 (ng/mL)	491.0 ± 132.8	493.9 ± 150.0	487.7 ± 110.9	0.706
MMP1 (pg/mL)	470.1 ± 1163.3	339.5 ± 550.5	614.0 ± 1573.7	0.689
MMP2 (ng/mL)	127.1 ± 40.8	124.0 ± 41.2	130.5 ± 40.1	0.053
MMP-9 (ng/mL)	142.0 ± 111.4	152.9 ± 114.9	129.8 ± 106.2	0.012
MCP1 (pg/mL)	73.4 ± 58.7	78.7 ± 66.7	67.5 ± 48.0	0.006
sTNFRII (pg/mL)	3262.4 ± 932.4	3322.7 ± 978.8	3195.9 ± 875.3	0.093
IL6 (pg/mL)	3.95 ± 7.28	4.00 ± 8.28	3.90 ± 5.98	0.127
Creatinine (mg/dL)	1.0 ± 0.5	1.1 ± 0.5	0.8 ± 0.4	<0.001
eGFR (mL/min/1.73 m^2^)	83.9 ± 20.6	86.7 ± 20.8	80.63 ± 19.8	0.001

BP, blood pressure; HDL, high-density lipoprotein; LDL, low-density lipoprotein; HOMA-IR, homeostasis model assessment of insulin resistance; QUICKI, quantitative insulin sensitivity check index; CRP, C-reactive protein; SAA, serum amyloid A; sE-selectin, soluble E-selectin; sP-selectin, soluble P-selectin; sICAM1, soluble intercellular adhesive molecule 1; sVCAM1, soluble vascular cell adhesive molecule 1; MMP-1, matrix metalloproteinase-1; MMP-2, matrix metalloproteinase-2; MMP-9, matrix metalloproteinase-9; MCP-1, monocyte chemotactic protein-1; sTNFR2, soluble tumor necrosis factor-alpha receptor 2; IL6, interleukin-6; eGFR, estimated glomerular filtration rate. Continuous variables are presented as mean ± standard deviation. HDL-C, LDL-C, total cholesterol, triglyceride, CRP, SAA, sICAM1, sVCAM1, sE-selectin, sP-selectin, MMP-1, MMP-2, MMP-9, YKL-40, MCP-1, and sTNFRII values were logarithmically transformed before statistical testing to meet the assumption of normal distributions; however, the untransformed data are shown. BP levels and lipid variables were analyzed with the exclusion of subjects using antihypertensive drugs and/or lipid-lowering agents, respectively. Fasting plasma glucose and insulin, QUICKI, and HOMA-IR index were analyzed with the exclusion of antidiabetic medications.

**Table 2:** Association between CRP and SAA levels and measurable risk factors in Taiwanese.

Clinical and biochemical parameters		CRP level				SAA level			
		*r*	*β* (95% CI)	*P* value^*∗*^	Adjusted *P* value	*r*	*β* (95% CI)	*P* value^*∗*^	Adjusted *P* value
Anthropology	Body mass index	0.318	0.045 (0.034–0.056)	1.9 × 10^−15^	6.1 × 10^−14^	0.121	0.017 (0.006–0.028)	0.003	0.096
	Waist-hip ratio	0.221	1.793 (1.157–2.430)	4.7 × 10^−8^	1.5 × 10^−6^	0.061	0.488 (−0.156–1.131)	0.137	NS

Blood pressure	Systolic BP	0.178	0.006 (0.003–0.008)	3.6 × 10^−5^	0.001	0.132	0.004 (0.001–0.007)	0.003	0.096
	Diastolic BP	0.152	0.008 (0.003–0.012)	4.4 ×10^−4^	0.014	0.141	0.007 (0.003–0.011)	0.001	0.032

Glucose metabolism	Fasting plasma glucose	0.139	1.056 (0.493–1.618)	0.001	0.032	0.002	0.001 (−0.568–0.570)	0.996	NS
	HOMA-IR index	0.299	0.778 (0.605–0.951)	1.2 × 10^−17^	3.9 × 10^−16^	0.138	0.335 (0.151–0.519)	0.001	0.032

Lipid profiles	LDL cholesterol	0.055	0.001 (0.000–0.002)	0.185	NS	0.032	<0.001 (−0.001–0.002)	0.449	NS
	HDL cholesterol	−0.246	−1.161 (−1.532 to −0.790)	1.4 × 10^−9^	4.6 × 10^−8^	0.074	0.345 (−0.036–0.725)	0.076	NS
	Triglyceride	0.267	0.528 (0.373–0.683)	5.0 × 10^−11^	1.6 × 10^−9^	0.095	0.186 (0.026–0.345)	0.022	0.704

Renal function	Urinary ACR	0.011	<0.001	0.783	NS	0.028	<0.001	0.501	NS
	Uric acid	0.227	0.077 (0.048–0.106)	3.8 × 10^−7^	1.2 × 10^−5^	0.131	0.043 (0.014–0.071)	0.004	0.128
	eGFR	0.196	0.006 (0.003–0.008)	1.1 × 10^−5^	3.5 × 10^−4^	0.030	0.001 (−0.002–0.003)	0.502	NS

Inflammatory markers	CRP	—	—	—	—	0.414	0.406 (0.334–0.479)	9.1 × 10^−26^	2.9 × 10^−24^
	SAA	0.414	0.423 (0.347–0.498)	9.1 × 10^−26^	2.9 × 10^−24^	—	—	—	—
	Fibrinogen	0.308	0.002 (0.002–0.003)	1.4 × 10^−14^	4.5 × 10^−13^	0.178	0.001 (0.001–0.002)	1.4 × 10^−5^	4.5 × 10^−4^
	sE-selectin	0.272	0.681 (0.486–0.877)	1.8 × 10^−11^	5.8 × 10^−10^	0.101	0.251 (0.051–0.451)	0.014	0.448
	sP-selectin	0.044	0.071 (−0.059–0.200)	0.287	NS	−0.004	−0.007 (−0.136–0.122)	0.916	NS
	sVCAM1	0.032	0.154 (−0.235–0.544)	0.436	NS	0.028	0.131 (−0.252–0.515)	0.502	NS
	sICAM1	0.126	0.331 (0.120–0.541)	0.002	0.064	0.019	0.049 (−0.160–0.258)	0.645	NS
	sTNFRII	0.180	0.753 (0.421–1.084)	1.0 × 10^−5^	3.2 × 10^−4^	0.073	0.299 (−0.034–0.631)	0.078	NS
	IL6	0.316	0.468 (0.372–0.564)	9.5 × 10^−15^	3.1 × 10^−14^	0.165	0.197 (0.102–0.291)	4.9 × 10^−5^	0.002
	MCP1	0.031	0.05 (−0.080–0.180)	0.452	NS	0.012	0.019 (−0.112–0.150)	0.772	NS
	MMP1	0.045	0.045 (−0.036–0.126)	0.275	NS	−0.002	−0.002 (−0.082–0.078)	0.959	NS
	MMP2	−0.119	−0.469 (−0.784 to −0.154)	0.004	0.128	−0.043	−0.235 (−0.548–0.078)	0.297	NS
	MMP9	0.077	0.146 (−0.007–0.299)	0.062	NS	0.076	0.145 (−0.008–0.297)	0.064	NS

Adipokines	Leptin	0.225	0.442 (0.325–0.559)	2.8 × 10^−8^	9.0 × 10^−7^	0.131	0.289 (0.169–0.409)	0.001	0.032
	Resistin	0.082	0.150 (0.000–0.301)	0.050	NS	−0.019	−0.034 (−0.183–0.116)	0.659	NS
	Lipocalin-2	0.136	0.310 (0.124–0.495)	0.001	0.032	0.085	0.190 (0.005–0.376)	0.044	NS
	Adiponectin	−0.251	−0.447 (−0.586 to −0.308)	5.3 × 10^−10^	1.7 × 10^−8^	−0.007	−0.013 (−0.155–0.129)	0.862	NS

^*∗*^ *P* value was adjusted for age and sex.

Adjust *P* values were computed by Bonferroni method, NS (not significant) if adjusted *P* value above 1.0.

Abbreviations are as in Table 1; BP levels, lipid variables, and uric acid levels were analyzed with the exclusion of subjects using antihypertensive drugs, lipid-lowering agents, and uric acid lowering agents, respectively. Fasting plasma glucose and insulin, QUICKI, and HOMA-IR index were analyzed with the exclusion of antidiabetic medications. Albumin-to-creatinine ratio (ACR) (mg/g) was analyzed with the exclusion of subjects with macroalbuminuria.

**Table 3:** Associations of the *CRP* and *SAA genotypes* with CRP and SAA levels.

	MM^*∗*^	Mm	mm	*P*1 value^*∗∗∗*^ (adjusted *P*)	*P*2 value (adjusted *P*)	MM	Mm + mm	*β* (95% CI)	*P*1 value (adjusted *P*)	*P*2 value (adjusted *P*)
*CRP* SNP and CRP level										
rs2794521	1.0 ± 1.3 (393)^*∗∗*^	1.2 ± 1.6 (176)	1.0 ± 1.1 (21)	0.987	NA	1.0 ± 1.3 (393)	1.2 ± 1.5 (197)	2.49 × 10^−5^ (−0.078–0.078)	0.999	NA
rs3091244^*∗∗∗∗*^	0.9 ± 1.2 (374)	1.5 ± 1.7 (39)	1.3 ± 1.6 (178)	4.94 × 10^−6^ (2.47 × 10^−5^)	NA	0.9 ± 1.2 (374)	1.4 ± 1.6 (217)	−0.179 (−0.253 to −0.105)	2.49 × 10^−6^ (1.25 × 10^−5^)	NA
rs1800947	1.1 ± 1.4 (512)	0.9 ± 1.5 (83)	0.3 ± 0.2 (2)	0.029	NA	1.1 ± 1.4 (512)	0.9 ± 1.5 (85)	0.115 (0.011–0.219)	0.030	NA
rs1130864	1.1 ± 1.4 (557)	1.4 ± 1.7 (39)	0.52 (1)	0.096	NA	1.1 ± 1.4 (557)	1.4 ± 1.6 (40)	−0.124 (−0.269–0.021)	0.093	NA
rs1205	0.8 ± 1.2 (197)	1.2 ± 1.4 (305)	1.3 ± 1.6 (88)	3.95 × 10^−5^ (1.98 × 10^−4^)	NA	0.8 ± 1.2 (*n* = 197)	1.2 ± 1.5 (*n* = 393)	−0.156 (−0.233 to −0.080)	6.28 × 10^−5^ (3.13 × 10^−4^)	NA
*CRP *SNP and SAA level										
rs2794521	5.6 ± 12.5 (386)	4.9 ± 8.1 (173)	4.6 ± 3.2 (21)	0.896	0.982	5.6 ± 12.5 (386)	4.8 ± 7.7 (194)	0.007 (−0.075–0.088)	0.873	0.951
rs3091244	5.8 ± 13.1 (370)	4.4 ± 2.9 (37)	4.6 ± 6.8 (174)	0.861	0.066	5.8 ± 13.1 (370)	4.5 ± 6.3 (211)	−0.014 (−0.093–0.065)	0.734	0.078
rs1800947	5.1 ± 10.1 (503)	7.0 ± 15.8 (82)	0.4 ± 0.7 (2)	0.997	0.358	5.1 ± 10.1 (503)	6.8 ± 15.7 (84)	−0.002 (−0.111–0.117)	0.969	0.339
rs1130864	5.4 ± 11.4 (548)	4.4 ± 3.0 (38)	1.97 (1)	0.694	0.698	5.4 ± 11.4 (548)	4.3 ± 2.9 (39)	−0.032 (−0.185–0.121)	0.683	0.705
rs1205	7.0 ± 17.2 (195)	4.3 ± 4.5 (298)	5.1 ± 9.1 (87)	0.906	0.059	7.0 ± 17.2 (195)	4.5 ± 5.9 (385)	0.005 (−0.076–0.086)	0.898	0.064
*SAA* SNP and SAA level										
rs4638289	3.0 ± 4.1 (237)	5.7 ± 10.4 (266)	11.3 ± 21.7 (75)	1.78 × 10^−26^ (7.12 × 10^−26^)	NA	3.0 ± 4.1 (237)	6.9 ± 13.8 (341)	−0.397 (−0.468 to −0.326)	1.87 × 10^−25^ (7.48 × 10^−25^)	NA
rs12218	5.9 ± 13.1 (304)	4.7 ± 8.5 (232)	5.5 ± 8.2 (39)	0.415	NA	5.9 ± 13.1 (304)	4.8 ± 8.4 (271)	−0.014 (−0.091–0.064)	0.726	NA
rs7131332	8.0 ± 14.5 (187)	4.4 ± 7.1 (292)	3.2 ± 12.6 (97)	1.45 × 10^−19^ (5.80 × 10^−19^)	NA	8.0 ± 14.6 (187)	4.1 ± 8.8 (389)	−0.338 (−0.415 to −0.261)	4.83 × 10^−17^ (1.93 × 10^−16^)	NA
rs11024591	7.9 ± 15.7 (211)	4.4 ± 7.7 (284)	2.0 ± 2.1 (82)	1.01 × 10^−20^ (4.04 × 10^−20^)	NA	7.9 ± 15.7 (*n* = 211)	3.8 ± 6.9 (*n* = 366)	—	3.21 × 10^−17^ (1.28 × 10^−16^)	NA
*SAA* SNP and CRP level										
rs4638289	1.1 ± 1.4 (245)	1.0 ± 1.4 (268)	1.3 ± 1.5 (75)	0.663	6.2 × 10^−6^ (2.48 × 10^−5^)	1.1 ± 1.4 (245)	1.1 ± 1.4 (343)	−0.013 (−0.088–0.062)	0.733	6.03 × 10^−6^ (2.41 × 10^−5^)
rs12218	1.0 ± 1.4 (308)	1.1 ± 1.3 (238)	1.5 ± 1.7 (39)	0.015	0.024	1.0 ± 1.4 (308)	1.1 ± 1.4 (277)	0.042 (−0.032–0.115)	0.267	0.133
rs7131332	1.2 ± 1.5 (188)	1.0 ± 1.4 (299)	0.9 ± 1.2 (99)	0.202	0.010	1.2 ± 1.5 (188)	1.0 ± 1.3 (398)	−0.087 (−0.165 to −0.009)	0.029	0.193
rs11024591	1.3 ± 1.5 (211)	1.0 ± 1.4 (291)	0.9 ± 1.2 (85)	0.251	0.004	1.3 ± 1.5 (211)	1.0 ± 1.3 (376)	−0.105 (−0.180 to −0.029)	0.007	0.439

^*∗*^M: major allele, m: minor allele, and ^*∗∗*^number of subjects below the CRP/SAA levels.

^*∗∗∗*^ *P*1: adjusted for age, sex, body mass index, smoking, and medications for hypertension, diabetes mellitus, and dyslipidemia, *P*2: adjusted for age, sex, body mass index, smoking, and medications for hypertension, diabetes mellitus, and dyslipidemia as well as CRP levels (for CRP genotypes) or SAA levels (for SAA genotypes). Adjusted *P*: Bonferroni correction for *P*1 or *P*2 values, and only significant *P* values were demonstrated.

^*∗∗∗∗*^rs3091244 is a triallelic locus (A, C, and T) and when the genotypes were analyzed separately, *P* value is 8.72 × 10^−5^ for CRP level and *P*1 and *P*2 values are 0.44 and 0.076, respectively, for SAA levels. The genotypes of rs3091244 were recoded as CC for MM, AA+AC+AT for Mm, and CT+TT for mm in the table [26].

**Table 4:** Associations of the *CRP* and *SAA* haplotypes with respective circulation levels in subjects from health examination.

Haplotypes	Frequency	CRP levels				SAA levels			
Gene		Coefficient	*P*1 value^*∗*^ (adjusted *P*)	Coefficient	*P*2 value (adjusted *P*)	Coefficient	*P*1 value (adjusted *P*)	Coefficient	*P*2 value (adjusted *P*)
*CRP*									
ACGCA	51.8%	−0.1601	2.66 × 10^−3^	NA	NA	0.0054	0.9230	0.0710	0.1684
GCGCG	18.3%	−0.0089	0.8973	NA	NA	0.0073	0.9184	0.0179	0.7855
AAGCG	16.0%	0.3120	1.447 × 10^−5^ (8.68 × 10^−5^)	NA	NA	−0.0535	0.4804	−0.1884	0.0077 (0.046)
ACCCA	7.0%	−0.2349	0.0268	NA	NA	−0.0252	0.8201	0.0692	0.4987
ATGTG	3.3%	0.2551	0.0809	NA	NA	0.0121	0.9371	−0.1074	0.4482
ACGCG	2.0%	0.2759	0.1290	NA	NA	0.1746	0.3547	0.0649	0.7090
*SAA*									
GATT	36.8%	−0.130	0.0189	0.1321	0.0213	−0.6147	6.92 × 10^−29^ (4.15 × 10^−28^)	NA	NA
AAAC	29.6%	0.0537	0.3601	−0.2507	3.18 × 10^−5^ (1.91 × 10^−4^)	0.6451	2.10 × 10^−28^ (1.20 × 10^−27^)	NA	NA
AGTC	24.5%	0.1145	0.0725	0.1208	0.0404	−0.0024	0.9712	NA	NA
GAAC	5.4%	0.0643	0.5872	−0.0020	0.9848	0.1499	0.2240	NA	NA
AGTT	1.9%	0.0830	0.6654	0.0247	0.8913	0.1920	0.3464	NA	NA

^*∗*^ *P*1 to *P*2 values: definitions as in Table 3.

For the adjusted *P* value, only significant *P* values were demonstrated, NA: not available.

**Table 5:** Mediation test of CRP and SAA levels in the association of *CRP* and *SAA1* genotypes/haplotypes with SAA and CRP levels.

	*SAA* variants for CRP level				*CRP* haplotype for SAA level
	rs4638289^#^	rs7131332^#^	rs11024591^#^	AAAC	AAGCG
*Criterion 1*					
*α*					
Regression coefficient	−0.040	−0.044	−0.047	0.645	0.312
Standard error	0.004	0.005	0.005	0.055	0.071
*P* value	3.87 × 10^−26^	1.79 × 10^−19^	7.37 × 10^−20^	2.10 × 10^−28^	1.45 × 10^−5^
*Criterion 2*					
*β*					
Regression coefficient	0.460	0.420	0.424	0.460	0.429
Standard error	0.041	0.040	0.040	0.041	0.040
*P* value	7.43 × 10^−27^	1.23 × 10^−23^	5.24 × 10^−24^	2.28 × 10^−26^	2.73 × 10^−24^
*γ*′					
Regression coefficient	0.017	0.012	0.014	−0.251	−0.188
Standard error	0.004	0.005	0.005	0.060	0.070
*P* value	1.24 × 10^−5^	0.014	0.007	3.18 × 10^−5^	0.008
*Criterion 3*					
*αβ* + *γ*′					
Regression coefficient	−0.002	−0.006	−0.006	0.054	−0.054
Standard error	0.004	0.005	0.005	0.059	0.076
*P* value	0.555	0.187	0.220	0.360	0.480
*Criterion 4*					
*αβ*					
Regression coefficient	−0.018	−0.018	−0.020	0.297	0.134
Standard error	0.002	0.003	0.003	0.037	0.033
*P* value (Sobel test)	<10^−8^	<10^−8^	<10^−8^	<10^−8^	4.78 × 10^−5^

^#^Represent *SAA *genotypes were analyzed in additive models.

*α*: unstandardized coefficient for the association between *SAA1* variants and log adiponectin levels or between *CRP* variants and log CRP levels.

*β*: unstandardized coefficient for the association between SAA and CRP levels.

Direct effect = *γ*′.

Total effect = *αβ* + *γ*′.

Mediation (indirect) effect = *αβ*.
