# Supplementary material for: CRP and SAA1 Haplotypes Are Associated with Both C-Reactive Protein and Serum Amyloid A Levels: Role of Suppression Effects
Source: Mediators Inflamm. 2016 May 25;2016:5830361. doi: 10.1155/2016/5830361 (PMC4897670; doi:10.1155/2016/5830361)
Supplement: Supplementary file 1 — Supplementary Materials include tables for the baseline data of the C-reactive protein (CRP) and serum amyloid A (SAA1) variants, linkage disequilibrium between the CRP and SAA1 variants and the association of the CRP and SAA1 variants with clinical parameters and other biomarker levels. [file 5830361.f1.doc]

Supplementary Table 1. Primer sequence of C-reactive protein (*CRP)* and serum amyloid A (*SAA1)* variants

| Gene | SNP number | Position | Location | Minor allele | MAF | HWE | Primer sequence | PCR size and RE |
| --- | --- | --- | --- | --- | --- | --- | --- | --- |
| *CRP* |  |  |  |  |  |  |  |  |
|  | rs2794521 | 159715306 | near 5’promoter | C | 0.187 | 1 | (F)5’-ACAACAGTCCCCAGAGTGTGATGTT-3’  (R)5’-ATGCTATGTCTGTGATCAGGCACAC-3’ | 224bp  NSPI |
|  | rs3091244 | 159714875 | near 5’promoter | T | 0.041 | 0.896 | (F)5’-ATTTCCCAGTCTGTAAATAAGCAAA-3’  (R)5’-AATGGGAAATGGTAACATATTAATC-3’ | 173bp  BfaI and TcqI |
|  | rs1800947 | 159713648 | exon2 | C | 0.073 | 0.893 | (F)-5'-GATCTGTGTGATCTGAGAAACCTCT-3' (R) 5'-GAGGTACCAGAGACAGAGACGTG -3' | 744bp  MaeIII |
|  | rs1130864 | 159713301 | exon2 | T | 0.033 | 1 | (F)-5'-AGCTCGTTAACTATGCTGGGGCA-3' (R)5'-CTTCTCAGCTCTT-GCCTTATGAGT-3' | 220bp  Bsp 1286I |
|  | rs1205 | 159712443 | exon2 | G | 0.408 | 0.481 | TaqMan SNP Genotyping Assays |  |
| *SAA1* |  |  |  |  |  |  |  |  |
|  | rs11024591 | 18263658 | near 3’ | T | 0.393 | 0.808 | TaqMan SNP Genotyping Assays |  |
|  | rs4638289 | 18264227 | near 3’ | A | 0.354 | 1 | TaqMan SNP Genotyping Assays |  |
|  | rs12218 | 18269774 | exon1 | G | 0.271 | 0.896 | TaqMan SNP Genotyping Assays |  |
|  | rs7131332 | 18273976 | near 5’promoter | G | 0.425 | 0.815 | TaqMan SNP Genotyping Assays |  |

SNP: single nucleotide polymorphism: MAF: minor allele frequency; HWE: Hardy-Weinberg equilibrium; PCR: polymerase chain reaction; RE: restriction enzyme

Supplementary Table 2. Linkage disequilibrium between *CRP* gene polymorphisms

|  | rs3091244 | rs2794521 | rs1800947 | rs1130864 | rs1205 |
| --- | --- | --- | --- | --- | --- |
| rs3091244 | - | D': 0.998 | D': 0.996 | D': 0.742 | D': 0.969 |
| rs2794521 | -  - | -  - | D': 0.957 | D': 0.694 | D': 0.989 |
| rs1800947 | -  - | -  - | -  - | D': 0.640 | D': 0.995 |
| rs1130864 | -  - | -  - | -  - | -  - | D': 0.998 |

Supplementary Table 3. Linkage disequilibrium between *SAA* gene polymorphisms

|  | rs7131332 | rs12218 | rs4638289 | rs10245291 |
| --- | --- | --- | --- | --- |
| rs7131332 | -  - | D': 1.000 | D': 0.647 | D': 0.881 |
| rs12218 | -  - | -  - | D': 0.941 | D': 0.846 |
| rs4638289 | -  - | -  - | -  - | D': 1.000 |

Supplementary table 4. Associations of the *SAA*-rs4638289 genotypes with clinical parameters and other biomarker levels

| Genotypes |  | *GG* | *GT* | *TT* | | *P*1 | *P*2 | *P*3 | *P*4 |
| --- | --- | --- | --- | --- | --- | --- | --- | --- | --- |
| Number |  | 75 | 268 | 245 | |  |  |  |  |
| Anthropology | Age (years) | 46.09 ± 10.42 | 47.19 ± 9.91 | 45.04 ± 9.78 | | 0.052 | 1 |  |  |
|  | Body mass index (kg/m2) | 24.48 ± 4.05 | 24.12 ± 3.12 | 24.46 ± 3.56 | | 0.164 | 1 |  |  |
|  | Waist circumference (cm) | 86.13 ± 11.08 | 84.6 ± 9.08 | 85.49 ± 9.48 | | 0.429 | 1 | 0.166 | 1 |
|  | Weight-hip ratio | 0.87 ± 0.07 | 0.87 ± 0.06 | 0.87 ± 0.06 | | 0.784 | 1 | 0.505 | 1 |
| Blood Pressure | Systolic BP (mmHg) | 114.20 ± 15.92 | 113.37 ± 15.93 | 112.49 ± 16.62 | | 0.677 | 1 | 0.298 | 1 |
|  | Diastolic BP (mmHg) | 74.82 ± 9.52 | 75.32 ± 9.54 | 74.65 ± 10.52 | | 0.623 | 1 | 0.472 | 1 |
| Glucose metabolism | Fasting plasma glucose (mg/dl) | 97.64 ± 26.06 | 96.81 ± 24.62 | 95.90 ± 19.41 | | 0.736 | 1 | 0.687 | 1 |
|  | Fasting serum insulin (μU/ml) | 9.13 ± 4.58 | 9.31 ± 4.88 | 9.21 ± 4.89 | | 0.554 | 1 | 0.350 | 1 |
|  | HOMA-IR index | 2.24 ± 1.37 | 2.26 ± 1.46 | 2.21 ± 1.36 | | 0.452 | 1 | 0.642 | 1 |
| Lipid profiles | Total cholesterol (mg/dL) | 200.34 ± 42.21 | 197.57 ± 35.02 | 199.57 ± 36.08 | | 0.450 | 1 | 0.024 | 1 |
|  | LDL-cholesterol (mg/dL) | 118.62 ± 41.21 | 113.72 ± 30.69 | 117.73 ± 32.26 | | 0.169 | 1 | 0.059 | 1 |
|  | HDL-cholesterol (mg/dL) | 55.63 ± 12.43 | 55.56 ± 15.62 | 54.67 ± 13.38 | | 0.442 | 1 | 0.622 | 1 |
|  | Triglyceride (mg/dL) | 130.81 ± 69.39 | 147.06 ± 131.42 | 142.32 ± 119.93 | | 0.900 | 1 | 0.267 | 1 |
| Renal function | Microalbumin/creatinine (ug/mg) | 9.64 ± 20.32 | 8.16 ± 9.49 | 9.85 ± 19.35 | | 0.329 | 1 | 0.052 | 1 |
|  | Creatinine (mg/dL) | 0.99 ± 0.22 | 1.00 ± 0.51 | | 0.98 ± 0.48 | 0.630 | 1 | 0.465 | 1 |
|  | eGFR (mL/min/1.73 m^2^) | 82.64 ± 17.69 | 81.79 ± 20.33 | 85.97 ± 21.33 | | 0.842 | 1 | 0.596 | 1 |
| Inflammation marker | CRP (mg/L) | 1.30 ± 1.49 | 1.02 ± 1.37 | 1.08 ± 1.37 | | 0.663 | 1 | 6.2 × 10^-6^ | 1.86 × 10^-5^ |
|  | Fibrinogen (mg/dL) | 275.63 ± 79.78 | 262.28 ± 66.62 | 258.63 ± 65.42 | | 0.252 | 1 | 0.463 | 1 |
|  | sE-selectin (ng/mL) | 48.59 ± 22.04 | 54.18 ± 28.67 | 53.44 ± 21.53 | | 0.154 | 1 | 0.024 | 1 |
|  | sP-selectin (ng/mL) | 131.27 ± 122.30 | 135.81 ± 107.77 | 146.01 ± 122.41 | | 0.126 | 1 | 0.206 | 1 |
|  | sVCAM1 (ng/mL) | 467.63 ± 101.50 | 510.46 ± 156.32 | 478.26 ± 110.38 | | 0.157 | 1 | 0.219 | 1 |
|  | sICAM1 (ng/mL) | 223.83 ± 66.69 | 243.81 ± 120.43 | 241.87 ± 112.10 | | 0.744 | 1 | 0.761 | 1 |
|  | sTNFRII (pg/mL) | 3283.9 ± 902.4 | 3270.5 ± 1006.1 | 3271.3 ± 861.8 | | 0.628 | 1 | 0.124 | 1 |
|  | IL6 (pg/mL) | 3.7 ± 6.4 | 3.8 ± 6.2 | 5.3 ± 12.3 | | 0.268 | 1 | 0.756 | 1 |
|  | MCP1 (pg/mL) | 76.21 ± 62.19 | 72.27 ± 56.09 | 74.21 ± 61.50 | | 0.459 | 1 | 0.456 | 1 |
|  | MMP1 (pg/mL) | 437.11 ± 967.78 | 438.00 ± 1140.80 | 528.30 ± 1264.57 | | 0.386 | 1 | 0.459 | 1 |
|  | MMP2 (ng/mL) | 134.49 ± 90.77 | 150.28 ± 129.64 | 135.15± 95.62 | | 0.228 | 1 | 0.100 | 1 |
|  | MMP9 (ng/mL) | 0.80 ± 0.43 | 0.59 ± 0.33 | 0.24 ± 0.51 | | 0.179 | 1 | 0.475 | 1 |
| Adipokines | Leptin (g/L) | 19.34 ± 16.74 | 17.45 ± 15.26 | 21.92 ± 23.33 | | 0.152 | 1 | 0.021 | 1 |
|  | Resistin (ng/mL) | 18.95 ± 13.41 | 19.01 ± 17.35 | 18.04 ± 11.02 | | 0.749 | 1 | 0.518 | 1 |
|  | Lipocalin2 (ng/mL) | 76.43 ± 33.54 | 84.20 ± 64.05 | 75.12 ± 39.72 | | 0.154 | 1 | 0.442 | 1 |
|  | Adiponectin (mg/L) | 7.09 ± 4.17 | 7.44 ± 5.77 | 6.92 ± 4.78 | | 0.433 | 1 | 0.828 | 1 |

Abbreviation as in Table1;

BP levels, lipid variables and uric acid levels were analyzed with the exclusion of subjects using antihypertensive drugs, lipid-lowering agents and uric acid lowering agents, respectively. Fasting plasma glucose and insulin, QUICKI and HOMA-IR index were analyzed with the exclusion of anti-diabetic medications.

Microalbumin/creatinine ratio (MCR) was analyzed with the exclusion of subjects with macroalbiminuria

*P*1: Adjusted for age, sex, body mass index, smoking and medications for hypertension, diabetes mellitus and dyslipidemia,

*P3* further adjustment of SAA levels

*P2* Bonferroni correction for *P1, P4* Bonferroni correction for *P3*

Supplementary table 5. Associations of the *CRP*-rs3091244 genotypes with clinical parameters and other biomarker levels

| Genotypes |  | *AA + AC + AT* | *CT + TT* | *CC* | | *P*1 | | *P*2 | *P*3 | *P*4 | | |
| --- | --- | --- | --- | --- | --- | --- | --- | --- | --- | --- | --- | --- |
| Number |  | 178 | 39 | 374 | |  | |  |  |  | | |
| Anthropology | Age (years) | 46.09 ± 9.30 | 46.50 ± 9.34 | 46.15 ± 10.36 | | 0.974 | | 1 |  |  | | |
|  | Body mass index (kg/m2) | 24.25 ± 3.55 | 24.69 ± 2.92 | 24.30 ± 3.42 | | 0.842 | | 1 |  |  | | |
|  | Waist circumference (cm) | 84.88 ± 9.81 | 87.85 ± 8.97 | 85.04 ± 9.38 | | 0.455 | | 1 | 0.205 | 1 | | |
|  | Weight-hip ratio | 0.87 ± 0.06 | 0.88 ± 0.05 | 0.87 ± 0.06 | | 0.902 | | 1 | 0.595 | 1 | | |
| Blood Pressure | Systolic BP (mmHg) | 112.42 ± 16.70 | 111.95 ± 12.54 | 113.55 ± 16.30 | | 0.564 | | 1 | 0.386 | 1 | | |
|  | Diastolic BP (mmHg) | 74.87 ± 10.71 | 74.11 ± 8.56 | 75.15 ± 9.72 | | 0.864 | | 1 | 0.750 | 1 | | |
| Glucose metabolism | Fasting plasma glucose (mg/dl) | 95.14 ± 14.40 | 102.44 ± 44.70 | 96.45 ± 22.51 | | 0.316 | | 1 | 0.151 | 1 | | |
|  | Fasting serum insulin (μU/ml) | 9.31 ± 5.23 | 9.82 ± 4.78 | 9.13 ± 4.64 | | 0.848 | | 1 | 0.599 | 1 | | |
|  | HOMA-IR index | 2.25 ± 1.51 | 2.63 ± 2.11 | 2.18 ± 1.25 | | 0.969 | | 1 | 0.472 | 1 | | |
| Lipid profiles | Total cholesterol (mg/dL) | 205.19 ± 36.65 | 197.28 ± 31.27 | 195.81 ± 36.55 | | 0.005 | | 0.15 | 0.010 | 0.3 | | |
|  | LDL-cholesterol (mg/dL) | 121.23 ± 33.54 | 113.36 ± 25.13 | 113.83 ± 33.07 | | 0.012 | | 0.36 | 0.019 | 0.57 | | |
|  | HDL-cholesterol (mg/dL) | 55.60 ± 13.76 | 52.18 ± 13.22 | 55.37 ± 14.73 | | 0.400 | | 1 | 0.132 | 1 | | |
|  | Triglyceride (mg/dL) | 147.76 ± 143.48 | 166.21 ± 136.87 | 137.35 ± 103.94 | | 0.947 | | 1 | 0.441 | 1 |  | |
| Renal function | Microalbumin/creatinine (ug/mg) | 9.41 ± 15.82 | 5.39 ± 3.74 | 9.22 ± 16.43 | | 0.523 | | 1 | 0.854 | 1 | | |
|  | Creatinine (mg/dL) | 0.96 ± 0.19 | 1.02 ± 0.18 | 1.00 ± 0.57 | 0.527 | | 1 | | 0.411 | 1 | |  |
|  | eGFR (mL/min/1.73 m^2^) | 83.47 ± 19.16 | 80.89 ± 17.88 | 84.23 ± 21.46 | | 0.800 | | 1 | 0.797 | 1 | | |
| Inflammation marker | Fibrinogen (mg/dL) | 262.54 ± 68.54 | 259.41 ± 73.61 | 263.27 ± 67.39 | | 0.982 | | 1 | 0.277 | 1 | | |
|  | sE-selectin (ng/mL) | 52.54 ± 21.80 | 58.22 ± 26.72 | 54.41 ± 25.03 | | 0.894 | | 1 | 0.549 | 1 | | |
|  | sP-selectin (ng/mL) | 135.29 ± 121.82 | 117.91 ± 112.00 | 142.24 ± 112.46 | | 0.923 | | 1 | 0.802 | 1 | | |
|  | sVCAM1 (ng/mL) | 482.88 ± 107.00 | 519.66 ± 102.46 | 492.39 ± 146.72 | | 0.415 | | 1 | 0.280 | 1 | | |
|  | sICAM1 (ng/mL) | 234.71 ± 97.24 | 247.64 ± 100.32 | 240.74 ± 116.68 | | 0.969 | | 1 | 0.719 | 1 | | |
|  | sTNFRII (pg/mL) | 3200.4 ± 921.2 | 3309.8 ± 788.3 | 3289.6 ± 350.9 | | 0.243 | | 1 | 0.066 | 1 | | |
|  | IL6 (pg/mL) | 4.9 ± 10.2 | 3.0 ± 3.4 | 3.6 ± 5.8 | | 0.169 | | 1 | 0.530 | 1 | | |
|  | MCP1 (pg/mL) | 71.31 ± 55.32 | 62.52 ± 43.14 | 75.65 ± 61.97 | | 0.204 | | 1 | 0.155 | 1 | | |
|  | MMP1 (pg/mL) | 493.0 ± 1440.0 | 223.5 ± 205.8 | 489.8 ± 1083.2 | | 0.527 | | 1 | 0.367 | 1 | | |
|  | MMP2 (ng/mL) | 123.77 ± 36.69 | 123.50 ± 33.86 | 129.31 ± 43.49 | | 0.183 | | 1 | 0.289 | 1 | | |
|  | MMP9 (ng/mL) | 141.82 ± 85.45 | 115.76 ± 75.62 | 114.36 ± 121.20 | | 0.952 | | 1 | 0.987 | 1 | | |
|  | SAA (mg/L) | 4.56 ± 6.80 | 4.42 ± 2.87 | 5.77 ± 13.05 | | 0.944 | | 1 | 0.076 | 1 | | |
| Adipokines | Leptin (g/L) | 17.66 ± 1.16 | 20.12 ± 16.53 | 20.39 ± 21.04 | | 0.113 | | 1 | 0.053 | 1 | | |
|  | Resistin (ng/mL) | 18.71 ± 12.97 | 18.01 ± 10.23 | 18.53 ± 15.46 | | 0.872 | | 1 | 0.882 | 1 | | |
|  | Lipocalin2 (ng/mL) | 78.57 ± 44.16 | 74.27 ± 28.63 | 80.48 ± 56.61 | | 0.724 | | 1 | 0.442 | 1 | | |
|  | Adiponectin (mg/L) | 6.82 ± 4.76 | 6.82 ± 4.60 | 7.41 ± 5.43 | | 0.476 | | 1 | 0.923 | 1 | | |

Abbreviation as in Table1; Subjects selected for analysis and *P* value definitions were similar to Supplementary table 4, with the exception of *P3* value further adjusted for CRP levels

Supplementary table 6. Associations of the *SAA*-rs12218 genotypes with clinical parameters and other biomarker levels

| Genotypes |  | *AA* | *AG* | *GG* | *P*1 | *P*2 | *P*3 | *P*4 |  | | |
| --- | --- | --- | --- | --- | --- | --- | --- | --- | --- | --- | --- |
| Number |  | 308 | 238 | 39 |  |  |  |  |  | | |
| Anthropology | Age (years) | 46.34 ± 10.0 | 45.94 ± 10.05 | 45.10 ± 9.10 |  |  |  |  |  | | |
|  | Body mass index (kg/m2) | 24.38 ± 3.36 | 24.26 ± 3.49 | 24.02 ± 3.69 |  |  |  |  |  | | |
|  | Waist circumference (cm) | 85.58 ± 9.53 | 85.05 ± 9.33 | 82.72 ± 10.44 | 0.183 | 1 | 0.159 | 1 | |  | |
|  | Weight-hip ratio | 0.87 ± 0.06 | 0.87 ± 0.07 | 0.84 ± 0.07 | 0.038 | 1 | 0.033 | 0.99 | |  | |
| Blood Pressure | Systolic BP (mmHg) | 113.23 ± 15.22 | 112.56 ± 16.88 | 115.0 ± 18.78 | 0.125 | 1 | 0.155 | 1 | |  | |
|  | Diastolic BP (mmHg) | 74.98 ± 9.77 | 74.59 ± 10.15 | 77.06 ± 10.15 | 0.031 | 0.93 | 0.041 | 1 | |  | |
| Glucose metabolism | Fasting plasma glucose (mg/dl) | 97.78 ± 25.63 | 96.01 ± 20.27 | 89.05 ± 7.69 | 0.068 | 1 | 0.065 | 1 | |  | |
|  | Fasting serum insulin (μU/ml) | 9.17 ± 4.56 | 9.41 ± 5.38 | 8.68 ± 3.16 | 0.784 | 1 | 0.671 | 1 | |  | |
|  | HOMA-IR index | 2.25 ± 1.43 | 2.26 ± 1.46 | 1.93 ± 0.77 | 0.321 | 1 | 0.260 | 1 | |  | |
| Lipid profiles | Total cholesterol (mg/dL) | 198.48 ± 36.14 | 197.56 ± 35.66 | 208.0 ± 41.39 | 0.057 | 1 | 0.078 | 1 | |  | |
|  | LDL-cholesterol (mg/dL) | 116.54 ± 33.87 | 114.03 ± 30.53 | 122.0 ± 37.14 | 0.149 | 1 | 0.179 | 1 | |  | |
|  | HDL-cholesterol (mg/dL) | 55.21 ± 14.98 | 54.89 ± 13.35 | 58.38 ± 15.47 | 0.754 | 1 | 0.812 | 1 | |  | |
|  | Triglyceride (mg/dL) | 137.10 ± 80.59 | 150.72 ± 157.66 | 144.79 ± 126.29 | 0.882 | 1 | 0.916 | 1 | | |  |
| Renal function | Microalbumin/creatinine (ug/mg) | 8.81 ± 16.20 | 8.16 ± 11.43 | 15.82 ± 29.02 | 0.009 | 0.27 | 0.011 | 0.33 | |  | |
|  | Creatinine (mg/dL) | 1.0 ± 0.45 | 0.96 ± 0.26 | 1.10 ± 1.16 | 0.018 | 0.54 | 0.020 | 0.60 | | | |
|  | eGFR (mL/min/1.73 m^2^) | 83.99 ± 20.49 | 83.95 ± 20.41 | 81.33 ± 18.38 | 0.126 | 1 | 0.139 | 1 | |  | |
|  | CRP (mg/L) | 1.04 ± 1.39 | 1.07 ± 1.32 | 1.53 ± 1.73 | 0.015 | 0.45 | 0.024 | 0.72 | |  | |
| Inflammation marker | Fibrinogen (mg/dL) | 265.90 ± 70.59 | 256.31 ± 65.74 | 274.31 ± 62.45 | 0.410 | 1 | 0.514 | 1 | |  | |
|  | sE-selectin (ng/mL) | 52.68 ± 25.0 | 54.64 ± 25.85 | 49.57 ± 21.79 | 0.912 | 1 | 0.936 | 1 | |  | |
|  | sP-selectin (ng/mL) | 139.04 ± 117.64 | 134.0 ± 103.74 | 174.04 ± 163.12 | 0.115 | 1 | 0.102 | 1 | |  | |
|  | sVCAM1 (ng/mL) | 487.36 ± 110.65 | 496.55 ± 160.49 | 495.62 ± 120.42 | 0.645 | 1 | 0.662 | 1 | |  | |
|  | sICAM1 (ng/mL) | 237.59 ± 103.81 | 244.60 ± 124.08 | 228.89 ± 89.23 | 0.506 | 1 | 0.509 | 1 | |  | |
|  | sTNFRII (pg/mL) | 3297.9 ± 990.1 | 3239.7 ± 873.2 | 3256.0 ± 907.2 | 0.976 | 1 | 0.967 | 1 | |  | |
|  | IL6 (pg/mL) | 3.7 ± 6.7 | 4.0 ± 8.0 | 5.3 ± 7.7 | 0.146 | 1 | 0.194 | 1 | |  | |
|  | MCP1 (pg/mL) | 74.33 ± 58.16 | 73.30 ± 62.80 | 67.38 ± 43.98 | 0.570 | 1 | 0.573 | 1 | |  | |
|  | MMP1 (pg/mL) | 437.8 ± 1062.1 | 485.9 ± 1278.5 | 731.7 ± 1371.1 | 0.016 | 0.48 | 0.015 | 0.45 | |  | |
|  | MMP2 (ng/mL) | 123.52 ± 33.64 | 131.27 ± 49.13 | 129.02 ± 31.34 | 0.484 | 1 | 0.480 | 1 | |  | |
|  | MMP9 (ng/mL) | 142.84 ± 106.55 | 144.67 ± 125.28 | 123.56 ± 59.61 | 0.365 | 1 | 0.331 | 1 | |  | |
| Adipokines | Leptin (g/L) | 18.67 ± 19.14 | 20.06 ± 18.01 | 23.60 ± 27.18 | 0.812 | 1 | 0.870 | 1 | |  | |
|  | Resistin (ng/mL) | 19.17 ± 16.67 | 17.81 ± 11.36 | 17.72 ± 11.51 | 0.582 | 1 | 0.591 | 1 | |  | |
|  | Lipocalin2 (ng/mL) | 80.32 ± 55.68 | 77.93 ± 42.24 | 80.87 ± 70.08 | 0.624 | 1 | 0.597 | 1 | |  | |
|  | Adiponectin (mg/L) | 7.79 ± 5.40 | 7.06 ± 5.03 | 7.99 ± 4.70 | 0.655 | 1 | 0.667 | 1 | |  | |

Abbreviation as in Table1;

Subjects selected for analysis and *P* value definitions were similar to Supplementary table 4

Supplementary table 7. Associations of the *CRP*-rs1205 genotypes with clinical parameters and other biomarker levels

| Genotypes |  | *GG* | *GT* | | *TT* | *P*1 | *P*2 | *P*3 | *P*4 |
| --- | --- | --- | --- | --- | --- | --- | --- | --- | --- |
| Number |  | 88 | 305 | | 197 |  |  |  |  |
| Anthropology | Age (years) | 46.48 ± 9.81 | 46.04 ± 9.67 | | 46.22 ± 10.55 |  |  |  |  |
|  | Body mass index (kg/m2) | 24.34 ± 3.57 | 24.45 ± 3.54 | | 24.07 ± 3.18 |  |  |  |  |
|  | Waist circumference (cm) | 85.07 ± 10.36 | 85.85 ± 9.30 | | 84.16 ± 9.38 | 0.181 | 1 | 0.402 | 1 |
|  | Weight-hip ratio | 0.87 ± 0.07 | 0.87 ± 0.06 | | 0.86 ± 0.07 | 0.201 | 1 | 0.382 | 1 |
| Blood Pressure | Systolic BP (mmHg) | 111.65 ± 16.22 | 114.19 ± 16.17 | | 111.98 ± 16.15 | 0.530 | 1 | 0.757 | 1 |
|  | Diastolic BP (mmHg) | 73.54 ± 9.10 | 75.64 ± 10.60 | | 74.58 ± 9.18 | 0.971 | 1 | 0.832 | 1 |
| Glucose metabolism | Fasting plasma glucose (mg/dl) | 95.73 ± 12.11 | 96.49 ± 24.14 | | 96.75 ± 24.03 | 0.664 | 1 | 0.387 | 1 |
|  | Fasting serum insulin (μU/ml) | 9.52 ± 4.67 | 9.31 ± 4.68 | | 9.0 ± 5.15 | 0.739 | 1 | 0.694 | 1 |
|  | HOMA-IR index | 2.30 ± 1.29 | 2.26 ± 1.45 | | 2.15 ± 1.39 | 0.635 | 1 | 0.777 | 1 |
| Lipid profiles | Total cholesterol (mg/dL) | 194.54 ± 32.47 | 200.14 ± 36.27 | | 198.11 ± 38.38 | 0.981 | 1 | 0.805 | 1 |
|  | LDL-cholesterol (mg/dL) | 112.85 ± 28.73 | 116.35 ± 32.71 | | 116.70 ± 34.86 | 0.602 | 1 | 0.452 | 1 |
|  | HDL-cholesterol (mg/dL) | 52.27 ± 14.22 | 54.29 ± 14.26 | | 55.40 ± 14.46 | 0.651 | 1 | 0.253 | 1 |
|  | Triglyceride (mg/dL) | 122.54 ± 96.87 | 152.63 ± 131.13 | | 134.81 ± 107.56 | 0.915 | 1 | 0.528 | 1 |
| Renal function | Microalbumin/creatinine (ug/mg) | 9.67 ± 12.82 | 9.17 ± 18.52 | | 8.53 ± 11.94 | 0.624 | 1 | 0.982 | 1 |
|  | Creatinine (mg/dL) | 0.95 ± 0.21 | 0.99 ± 0.25 | 1.01 ± 0.73 | | 0.393 | 1 | 0.288 | 1 |
|  | eGFR (mL/min/1.73 m^2^) | 83.53 ± 19.31 | 83.57 ± 20.01 | | 84.12 ± 21.97 | 0.259 | 1 | 0.253 | 1 |
| Inflammation marker | Fibrinogen (mg/dL) | 264.17 ± 68.76 | 259.63 ± 65.87 | | 267.09 ± 71.24 | 0.205 | 1 | 0.012 | 0.360 |
|  | sE-selectin (ng/mL) | 50.66 ± 21.38 | 55.40 ± 26.66 | | 49.87 ± 20.89 | 0.158 | 1 | 0.497 | 1 |
|  | sP-selectin (ng/mL) | 129.44 ± 103.30 | 138.66 ± 118.47 | | 142.62 ± 115.92 | 0.408 | 1 | 0.318 | 1 |
|  | sVCAM1 (ng/mL) | 495.02 ± 99.02 | 489.92 ± 154.56 | | 491.79 ± 110.12 | 0.708 | 1 | 0.518 | 1 |
|  | sICAM1 (ng/mL) | 243.95 ± 114.26 | 239.11 ± 111.08 | | 237.43 ± 107.07 | 0.955 | 1 | 0.783 | 1 |
|  | sTNFRII (pg/mL) | 3223.0 ± 932.8 | 3279.0 ± 924.9 | | 3273.2 ± 948.1 | 0.644 | 1 | 0.250 | 1 |
|  | IL6 (pg/mL) | 3.1 ± 5.9 | 4.8 ± 9.2 | | 3.0 ± 3.5 | 0.106 | 11 | 0.590 | 1 |
|  | MCP1 (pg/mL) | 65.86 ± 45.08 | 74.88 ± 58.62 | | 74.68 ± 64.81 | 0.411 | 1 | 0.328 | 1 |
|  | MMP1 (pg/mL) | 481.3 ± 1275.7 | 398.1± 1015.3 | | 587.3 ± 1334.3 | 0.101 | 1 | 0.052 | 1 |
|  | MMP2 (ng/mL) | 127.02 ± 30.21 | 129.34 ± 46.62 | | 124.09 ± 35.55 | 0.152 | 1 | 0.078 | 1 |
|  | MMP9 (ng/mL) | 144.93 ± 111.65 | 142.37 ± 112.03 | | 139.12 ± 111.57 | 0.877 | 1 | 0.912 | 1 |
|  | SAA (mg/L) | 5.07 ± 9.05 | 4.30 ± 4.53 | | 6.99 ± 17.2 | 0.906 | 1 | 0.059 | 1 |
| Adipokines | Leptin (g/L) | 21.60 ± 20.70 | 19.22 ± 16.93 | | 19.15 ± 21.87 | 0.868 | 1 | 0.863 | 1 |
|  | Resistin (ng/mL) | 18.91 ± 13.41 | 18.54 ± 12.22 | | 18.44 ± 17.75 | 0.422 | 1 | 0.600 | 1 |
|  | Lipocalin2 (ng/mL) | 88.30 ± 51.07 | 76.09 ± 39.0 | | 80.09 ± 66.55 | 0.967 | 1 | 0.731 | 1 |
|  | Adiponectin (mg/L) | 7.30 ± 5.15 | 6.76 ± 4.57 | | 7.82 ± 6.0 | 0.355 | 1 | 0.774 | 1 |

Abbreviation as in Table1; Subjects selected for analysis and *P* value definitions were similar to Supplementary table 5
